# Efficient inhibition of HIV-1 replication by an artificial polycistronic miRNA construct

**DOI:** 10.1186/1743-422X-9-118

**Published:** 2012-06-18

**Authors:** Tao Zhang, Tong Cheng, Lihua Wei, Yijun Cai, Anthony Et Yeo, Jiahuai Han, Y Adam Yuan, Jun Zhang, Ningshao Xia

**Affiliations:** 1 National Institute of Diagnostics and Vaccine Development in Infectious Diseases, Research Center for Medical Molecular Virology of Fujian Province, School of Life Science, Xiamen University, Xiamen, 361005, People’s Republic of China; 2 The Key Laboratory of the Ministry of Education for Cell Biology and Tumor Cell Engineering, School of Life Science, Xiamen University, Xiamen, 361005, People’s Republic of China; 3 Department of Biological Sciences and Temasek Life Sciences Laboratory, National University of Singapore, Singapore, 117543, Singapore; 4 Xiamen-National University of Singapore Joint Laboratory in Biomedical Sciences, Xiamen University, Xiamen, 361005, People’s Republic of China

**Keywords:** Artificial polycistronic transcript, HIV replication inhibition, Viral escape, RNA interference, siRNA

## Abstract

**Background:**

RNA interference (RNAi) has been used as a promising approach to inhibit human immunodeficiency virus type 1 (HIV-1) replication for both *in vitro* and *in vivo* animal models. However, HIV-1 escape mutants after RNAi treatment have been reported. Expressing multiple small interfering RNAs (siRNAs) against conserved viral sequences can serve as a genetic barrier for viral escape, and optimization of the efficiency of this process was the aim of this study.

**Results:**

An artificial polycistronic transcript driven by a CMV promoter was designed to inhibit HIV-1 replication. The artificial polycistronic transcript contained two pre-miR-30a backbones and one pre-miR-155 backbone, which are linked by a sequence derived from antisense RNA sequence targeting the HIV-1 *env* gene. Our results demonstrated that this artificial polycistronic transcript simultaneously expresses three anti-HIV siRNAs and efficiently inhibits HIV-1 replication. In addition, the biosafety of MT-4 cells expressing this polycistronic miRNA transcript was evaluated, and no apparent impacts on cell proliferation rate, interferon response, and interruption of native miRNA processing were observed.

**Conclusions:**

The strategy described here to generate an artificial polycistronic transcript to inhibit viral replication provided an opportunity to select and optimize many factors to yield highly efficient constructs expressing multiple siRNAs against viral infection.

## Background

RNA interference (RNAi) is a sequence-specific post-transcriptional gene-silencing mechanism, that was first discovered in *Caenorhabditis elegans*[1]. RNAi can be triggered by small interfering RNAs (siRNAs) or endogenous microRNAs (miRNAs), which are processed by RNase III-like enzymes [2,3]. Most miRNAs are processed from longer primary miRNA transcripts (pri-miRNA), which are transcribed from genome sequence by RNA polymerase II promoter [4,5]. Subsequently, pri-miRNAs are processed into miRNA precursors (pre-miRNAs) that are approximately 60 nucleotides (nt) long by miRNA processing machinery consisting of the nuclear Drospha-DGCR8 complex [6,7]. Next, pre-miRNAs are transported to the cytoplasm by Exportin-5 and further processed by Dicer to produce miRNA duplexes of approximately 22 nt in length [8,9]. The miRNA duplexes are loaded into the RNA-induced silencing complex (RISC) to guide RISC-mediated gene regulation via mRNA cleavage or translational repression [10,11]. Notably, several miRNA genes are encoded as clusters within the genome sequence and transcribed into pri-miRNAs simultaneously as clusters[12]. Hence, multiple miRNAs can be transcribed and processed from a single transcription unit [13,14].

Regarded as a potent post-transcription gene silencing tool, RNAi is now used as a standard laboratory tool to knock down gene expression at the cellular level, as well as at the organismal level [15,16]. In addition, RNAi has been successfully used as a promising approach to inhibit the replication of different viruses, including human immunodeficiency virus type 1 (HIV-1) [17-22]. Many strategies have been proposed to inhibit HIV-1 replication in cell culture and animal models, including siRNA or short hairpin RNA (shRNA) vector-based or pri-miRNA vector-based approaches [23-25]. These vector-based approaches have demonstrated long-term inhibition of HIV replication. However, due to the restriction of the RNAi mechanism (sequence-specificity) and high mutation rate of HIV-1, escape mutants after RNAi treatment have been reported [26-28]. Therefore, a combination of multiple antiviral inhibitors to overcome escape has been proposed.

Currently, both multiple shRNAs in a combinatorial vector approach and multiple antiviral siRNAs embedded in a single polycistronic miRNA transcript approach have been used to reduce the chance of viral escape [29-32]. Of these approaches, polycistronic miRNA has been shown to be safer, since the expression of miRNA-like transcripts is low and regulated, therefore reducing the risk of toxicity [33].

Native miRNA clusters and tandem copes of miR155 have been employed as the basis for the design of a polycistronic transcript that simultaneously expresses multiple antiviral siRNAs [29,31,32]. The replacement of the mature miRNA sequence by the miRNA-like stem will produce mature siRNAs that specifically target viruses. Several studies have shown that the native flanking pri-miRNA sequences and key structural features of the native miRNAs were retained, as they were thought to be critical for efficient siRNA processing [31].

In this study, we designed an artificial polycistronic transcript containing two pre-miR-30a backbones and one pre-miR-155 backbone, which is driven by a cytomegalovirus (CMV) promoter. Moreover, we used antisense RNA sequence targeting the HIV-1 *env* gene as the linker to connect the pre-miRNA backbones. This study demonstrated that the flanking pri-miRNA sequence can be replaced and optimized with artificial sequence to construct the polycistronic transcript that expresses three anti-HIV siRNAs simultaneously and efficiently inhibits HIV-1 replication. This strategy provides a feasible method to replace the flanking pri-miRNA sequences with other antiviral elements to design more complicated and efficient inhibitors against pathogens that are prone to escape.

## Results

### Screening of shRNA constructs inhibiting HIV-1 replication

**Figure 1:**
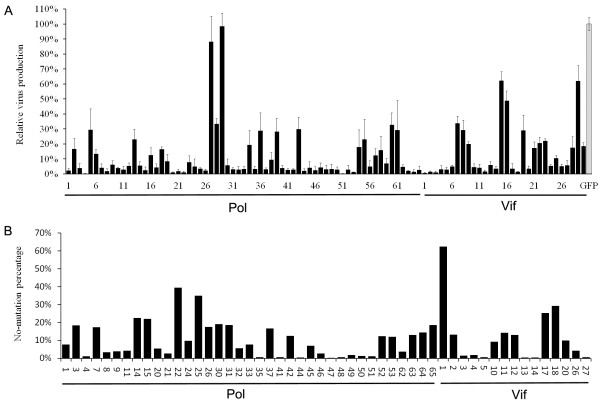
**Screening of highly effective shRNAs against HIV-1 sequences.** (**A**) The relative virus production (black bars) in a cotransfection assay of indicated shRNAs with HIV-1 infectious clone, pNL4-3. Virus production in the presence of shRNA targeting GFP was used as a negative control (gray bar). Virus production was measured by CA-p24 ELISA and corrected for transfection efficiency by including Renilla luciferase in the transfection assay. The ratio between CA-p24 and Renilla values yielded the relative virus production, which was set at 100% for the negative control. The mean values and standard deviations were obtained from three independent transfections. (**B**) The 52 shRNA constructs with inhibition efficiency greater than 97% were aligned with 625 HIV-1 full sequences in the Los Alamos HIV Sequence Database. The non-mutation percentage of shRNA target sequence is shown here.

To generate highly efficient constructs that inhibit HIV-1 replication, we employed a traditional shRNA-vector based approach to screen the best siRNA candidates to inhibit HIV-1 replication. A total of 95 shRNA constructs were developed, assisted by online design tools, to specifically target *pol* and *vif* transcripts. The *pol* gene encodes the key enzyme involved in HIV-1 replication and *vif* plays a role in disrupting the antiviral activity of the human enzyme APOBEC. Among the constructs, 65 shRNA constructs targeted *pol* and 30 shRNA constructs target *vif*. Each shRNA expression construct was cotransfected with the pNL4-3 infectious molecular clone to determine its ability to inhibit virus production. Fifty-two shRNA constructs with inhibition efficiency >97% were selected (Figure 1A). The HIV-1 sequence targeted by these selected shRNA constructs were aligned with 625 HIV-1 full-length sequences in the Los Alamos HIV Sequence Database. The three most conserved siRNA sequences yielded from the shRNA constructs (Pol22 (A), Pol25 (B) and Vif1 (C)) were selected for artificial miRNA transcript construction (Figure 1B).

### Construction of single antiviral miRNA transcripts

**Figure 2:**
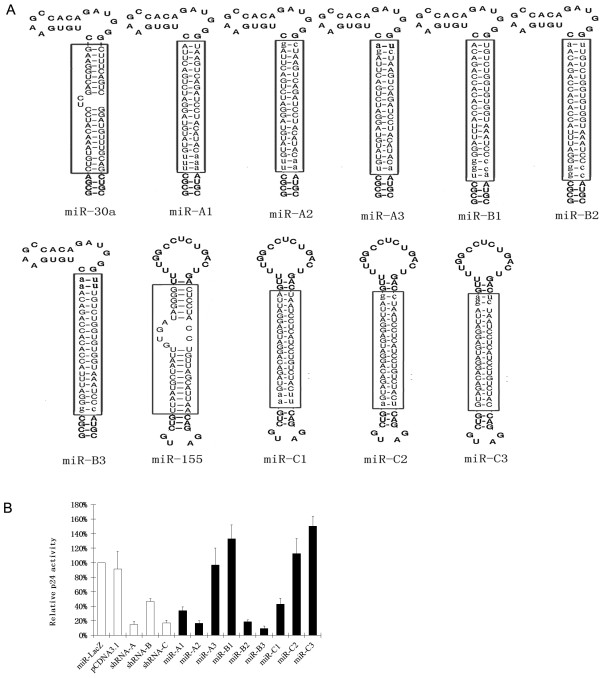
**Construction of the antiviral miRNA transcripts.** (**A**) The siRNAs A and B were extended and inserted into the miR-30a backbone; siRNA C was extended and inserted into the miR-155 backbone. Bulges that existed in mature wild-type miRNA were eliminated in antiviral miRNAs. Extended sequences in 22 bp siRNAs are in lowercase, whereas native mature miRNA sequence or inserted siRNA sequences are boxed. (**B**) Screening of effective antiviral miRNAs. The 239FT cells were co-transfected with 500 ng HIV-1 molecular clone pNL4-3, 50 ng pRL, and 100 ng miRNA constructs. The miR-LacZ against β-galactosidase was used as the negative control, whereas shRNAs expressing original 19 bp siRNA was the positive control. CA-p24 levels in the culture supernatant were measured 2 days post-transfection. CA-p24 expression in the presence of miR-LacZ was normalized at 100%. Renilla luciferase was used for normalization of transfection efficiency. Error bars represent the standard deviation of three independent experiments.

Selected pol22 (A), pol25 (B) and vif1 (C) siRNAs were extended from 19 bp to 22 bp by adding the adjacent sequences of the HIV-1_NL4_ target sites to the ends. To generate the artificial miRNA transcripts, the extended pol22 (A), pol25 (B) and vif1 (C) siRNAs were used to replace the mature miRNA sequences embedded in the pre-miR-30a or miR-155 backbones that would be suitable for artificial miRNA construction[34,35] (Figure 2A). The artificial miRNA transcripts were inserted into the pcDNA3.1 plasmid driven by a CMV promoter.

To determine the inhibitory efficiency, the artificial miRNA expressing plasmids were cotransfected with pNL4-3. Artificial miR-LacZ against β-gal-expressing plasmids was used as negative control, whereas the original shRNA vectors were used as a positive control. Among the various artificial miRNA constructs screened, miR-A2, miR-B3, and miR-C1 had the most inhibition activity against HIV-1 replication (Figure 2B).

### Construction of artificial polycistronic miRNA transcripts

**Figure 3:**
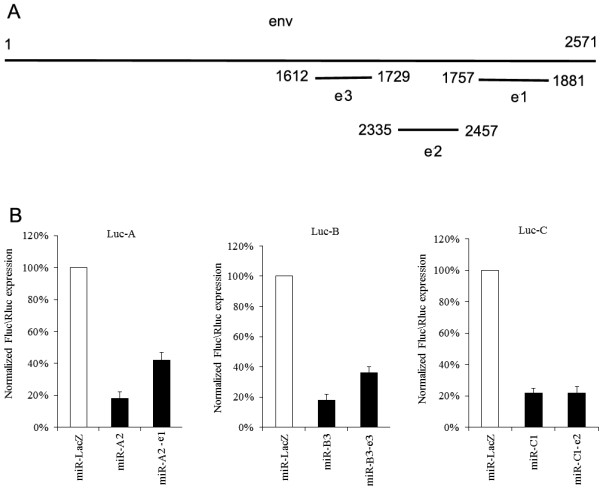
**Construction of the basic structural elements.** (**A**) Antisense RNAs targeted to HIV-1 *env* were used as linkers, and the positions in the *env* gene are indicated. (**B**) Inhibition of the structure elements. Firefly luciferase activity in the cell was measured 2 days post-transfection and normalized to the Renilla luciferase activity. Firefly luciferase activity in the presence of miR-LacZ was normalized at 100%.

In the miR-17-92 cluster, the linker length between two adjacent stem-loop structures was approximately 130 bp and the antisense RNA sequences targeted to the HIV-1 may have a lesser side-effect. Therefore, three RNA sequences 130 bp in length and complementary to the HIV-1 *env* sequences, named e1, e2 and e3, were employed as linker sequences (Figure 3A).

In the constructs, e1, e2 or e3 was added to the downstream region of selected miR-A2, miR-C1 or miR-B3, respectively, for individual miR-A2-e1, miR-C1-e2, and miR-B3-e3 transcripts. Determination of the inhibitory activities of these basic structural elements was evaluated by the firefly luciferase reporter assay. Firefly luciferase expression was normalized to the Renilla luciferase expression from the co-transfected pRL plasmid. These three basic constructs were able to inhibit the expression of the reporter gene, although the inhibitory efficiency of miR-B3-e3 and miR-A2-e1 decreased by approximately 50% (Figure 3B).

To investigate whether linkers exerted anti-HIV-1 activity, plasmids expressing linkers only were co-transfected with pNL4-3. Our data demonstrated that linkers exhibited little antiviral activity (Additional file 1: Figure S1), which is consistent with the observation that antisense RNA shorter than 400 nucleotides is incapable of inhibiting HIV-1 replication[36]. The individual artificial miRNA transcripts were then ligated to construct artificial polycistronic miRNA transcripts, which were named for the miRNA transcript followed by the linker name. For example, miR-AB stands for polycistronic miRNA transcript miR-A2-e1 connected by polycistronic miRNA transcript miR-B3-e3 successively, whereas miR-BA stands for miR-B3-e3 connected by miR-A2-e1 successively (Additional file 1: Figure S2).

**Figure 4:**
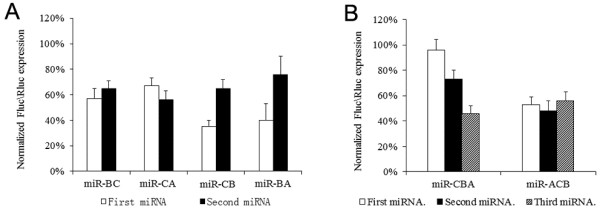
**Screening of the combinations of the basic structural elements.** The miRNA complexes were co-transfected with luciferase reporters in 293FT cells. Luciferase activity in cells was measured 48 h post-transfection and normalized to the Renilla luciferase activity. miR-LacZ was used as a negative control. The relative luciferase activity was defined according the ratio of individual miRNA to miR-LacZ. (**A**) Screening of the combinations of two basic structural elements. (**B**) Screening of the combinations of three basic structural elements. The combination of miR-CB with miR-A produces 2 varieties of triple miRNA clusters: miR-ACB and miR-CBA. The inhibitory effect of miR-ACB was greater than that for miR-CBA.

Polycistronic miRNA transcripts containing plasmids were co-transfected with luciferase reporter vectors into 293FT cells to measure the gene knockdown efficiency. A single artificial miR-LacZ transcript was used as negative control. The relative luciferase activity for the individual polycistronic miRNA transcript was calculated against that of the miR-lacZ transcript. Hence, the relative luciferase activity of miR-AB was defined as the miR-AB activity divided by miR-LacZ activity. The miR-CB construct displayed the highest inhibition efficiency among the bicistronic miRNA transcripts (Figure 4A). Next, we constructed two triple cistronic miRNA transcripts (miR-ACB and miR-CBA) and tested their suppression activities. Among these, miR-ACB showed better suppression activity than that for miR-CBA (Figure 4B). The inhibition efficiency of miRNA constructs were lower as compared to that shown by other studies [29,31]. Lo and Stegmeier have shown that the addition of a *gfp* gene between the promoter and the miRNA sequence significantly increases the inhibition efficiency of the miRNA [34,37]. Therefore, we attempted a similar technique by placing the gene between the promoter and the miRNA constructs.

### Construction of MT-4 cells expressing polycistronic miRNA transcripts

**Figure 5:**
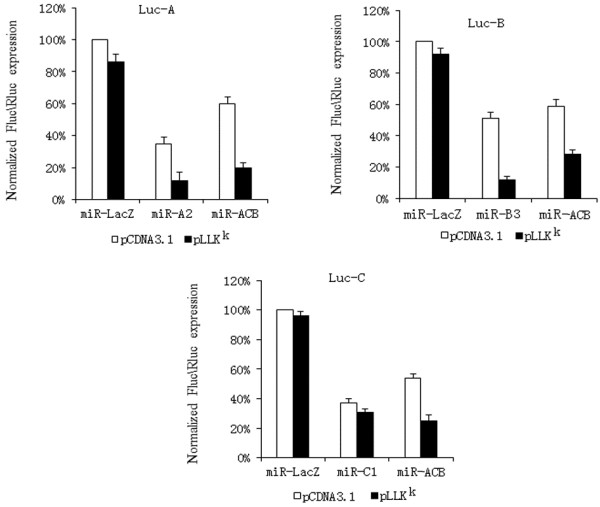
**Inhibitory efficiency of miR-ACB in the Lentiviral vector.** Inhibitory efficiency of each miRNA element in miR-ACB was measured by luciferase reporter assays. The miRNA encoding plasmids based on pcDNA3.1 or pLLK^K^ were co-transfected with luciferase reporters and activities in cells were measured 48 h post-transfection.

The lentiviral transfer plasmid, pLLK^k^, contained two genes encoding enhanced GFP (EGFP) and *H-2K*^*k*^. To increase the inhibition efficiency, selected antiviral tri-cistronic miRNA transcripts were inserted into the 3′-untranslated region (UTR) of the *H-2K*^*k*^ gene. Evaluation of the inhibitory efficiency of each of the antiviral miRNAs embedded in pLLK^k^ was measured using the luciferase reporter system. These data show that miR-ACB embedded in the pLLK^k^ vector displayed higher inhibition activity against three luciferase reporter gene expression tests than that embedded in the pcDNA3.1 vector (Figure 5). As expected, the tri-cistronic miRNA transcript miR-ACB exhibited higher suppression efficiency against the production of HIV-1_NL4-3_ virus than any single miRNA transcript (Additional file 1: Figure S3).

### Mature miRNA levels of miR-ACB in MT-4

**Figure 6:**
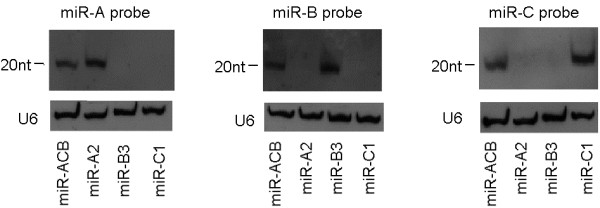
**Expression levels of mature miRNAs derived from miR-ACB transcripts compared to individual antiviral miRNA transcripts.** RNA was isolated from MT-4 cell lines expressing miRNAs and analyzed by northern blot using 19 nt complementary oligonucleotide probes. U6 RNA served as a loading control.

Recombinant lentiviruses encoding miRNAs transcripts were used to transduce MT-4 cells. To investigate whether the significant increase of the inhibition activity against HIV-1 replication by the artificial tri-cistronic miRNA transcript was due to the presence of multiple miRNAs that act synergistically to inhibit HIV-1 replication, miRNA expression levels in MT-4 cells was determined by northern blot. As showed in Figure 6, miR-ACB was successfully processed into three mature miRNAs and the amounts of these miRNAs were similar to those from pre-miRNA containing only one antiviral miRNA.

### Evaluation of off-target effects of miR-ACB

**Figure 7:**
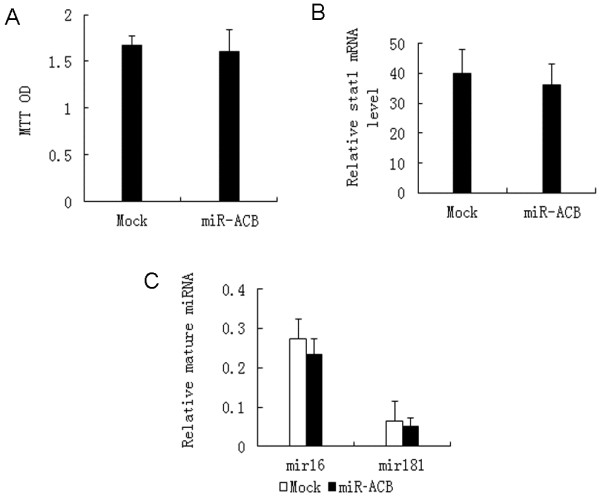
**Evaluation of off-target effects of miR-ACB.** (**A**) The cell proliferation rate of MT-4 cells with and without miR-ACB. MTT was detected after cells were plated and cultured 48 h at 37 °C. (**B**) The Stat1 mRNA level of MT-4 cells with and without miR-ACB was quantified by qPCR and normalized to the β-actin mRNAs. (**C**) Expression profiles of miRNAs in MT-4 with and without miR-ACB. Expression level of miRNAs was measured by qPCR and normalized to that of small non-coding RNA U6B. Error bars in all cases indicate the standard deviation of triplicate cultures.

As a first step to evaluate the biosafety of MT-4 cells expressing miR-ACB, three aspects, namely cell proliferation rate, interferon response, and interruption of native mRNA processing, were assessed in MT-4 cells with and without miR-ACB transcript expression. MTT was used to measure the cell proliferation. As showed in Figure 7A, the expression of the miR-ACB transcript had no effect on cell proliferation. Similarly, the protein expression level of Stat1, an interferon-stimulated transcription activator, was assayed. As shown in Figure 7B, the expression of miR-ACB transcript had no effect on Stat1 expression, which suggests expression of miR-ACB does not trigger the production of interferon. Finally, there were no alterations in the expression levels of miR-16 and miR-181 accompanying the expression of miR-ACB transcripts in MT-4 cells (Figure 7C).

### Potential prevention of HIV-1 escape by miR-ACB

**Figure 8:**
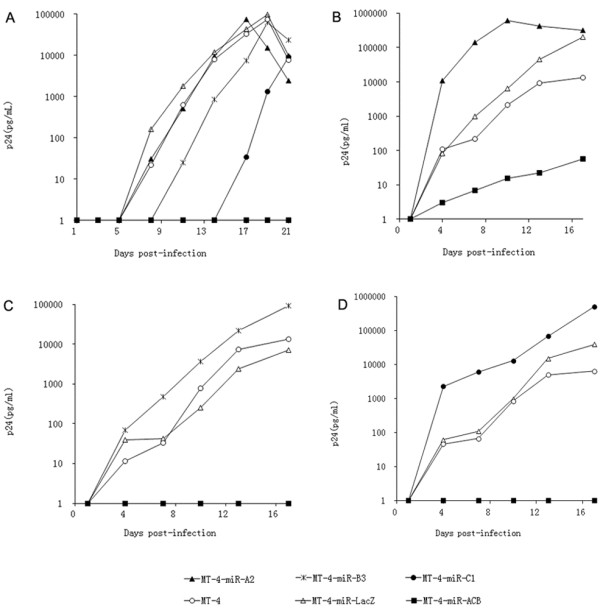
**Inhibition of virus replication in MT-4 cells by miR-ACB.** MT-4 cell lines expressing antiviral genes were infected with HIV-1. Viral replication was monitored by measuring the expression of CA-p24. Values for CA-p24 concentration are averages of three independent experiments. (**A**) Wild-type HIV-1_NL4-3_ virus; (**B**) miR-A2 resistant virus; (**C**) miR-B3 resistant virus; (**D**) miR-C1 resistant virus. MT-4 cells and MT-4-miR-LacZ were used as negative controls.

To investigate whether miR-ACB has the ability to prevent HIV-1 escape, MT-4 cells expressing miR-A2, miR-B3, miR-C1 or miR-ACB transcripts were infected with HIV-1_NL4-3_. MT-4 cells and MT-4 cells expressing miR-LacZ transcript were used as negative control. As shown in Figure 8A, although virus replication was initially inhibited by miR-A2, miR-B3, and miR-C1, in long term culture the replication of viruses rebounded. In contrast, MT-4 expressing miR-ACB transcript was able to inhibit HIV-1 replication for 21 days. In order to confirm these results, escaped viruses were collected from the supernatant of the MT-4 cells expressing single miRNA transcript 19 days post-infection and used to re-infect new MT-4 cells. Resistant virus collected from the miR-A2 cell supernatant replicated in MT-4 cells, MT-4-miR-LacZ cells, and MT-4-miR-A2 cells with similar growth kinetics, but were inhibited in the miR-ACB cells (Figure 8B). Similar results were observed for viruses resistant to miR-B3 and miR-C1 inhibition (Figures 8C-D). Thus, miR-ACB showed better inhibitory efficiency than individual antiviral miRNA transcripts, and has the potential to delay the emergence of resistant virus.

## Discussion

The selection and construction of cell lines expressing multiple effective antiviral elements are critical to avoid escape mutations in HIV-1 by RNAi techniques. Expressing multiple shRNAs from separate promoters and long hairpin RNAs has been reported to achieve inhibition of viral replication [30,38,39]. However, the high expression level of RNA polymerase III promoters that are used to transcribe shRNAs and lhRNAs may increase toxicity due to saturation of the RNAi machinery [33,40]. Another attractive approach is to express multiple antiviral siRNAs from a single polycistronic miRNA transcript that can be expressed from a single RNA polymerase II promoter to allow lower and regulated expression [29,31]. The native miRNA clusters (mir-17-92 or mir-106b) were used as the backbone for insertion of multiple antiviral siRNAs. It was reported that the native flanking primary miRNA (pri-miRNA) sequences are maintained to keep the structural features of the native miRNAs, which are critical for efficient siRNA processing [31]. In this study, we investigated a method to link multiple antiviral miRNA against HIV-1 with artificial flanking pri-miRNA sequence.

Firstly, we employed a conventional antiviral shRNA approach to select the antiviral small RNA sequences specifically targeting the two different HIV-1 targets, *pol* and *vif*. Secondly, the three selected small RNA sequences (pol22, pol25, and vif1 from 95 different sequences) were modified and ligated into the respectively pre-miR-30a or miR-155 backbones to generate three different artificial miRNA transcripts (miR-A2, miR-B3 or miR-C1). The individual miRNA transcripts were linked by arbitrary antisense RNA sequences targeted to HIV-1 *env* to construct an artificial single cistronic miRNA construct. Finally, the relative positions and the combinations of these artificial miRNA transcripts, together with the antisense RNA linker, were optimized based on a luciferase reporter system to acquire a tri-cistronic miRNA transcript (miR-ACB) with high expression levels of three individual antiviral siRNAs simultaneously. As a result, the MT-4 cells expressing the selected miR-ACB transcript exerted stronger inhibition of HIV-1 replication than any of the single antiviral miRNAs and had better suppressive activity against escaped virus replication than individual miRNA transcripts. Off-target effects of miRNA on cellular transcripts with partial sequence complementarity may induce negative effects on the treated cell. In this study, we did not observed any off-target effects of miR-ACB by evaluating cell proliferation rate, interferon response, and interruption of native mRNAs.

The strategy described here provides a method to construct miRNA polycistrons with artificial flanking pri-miRNA sequences. The results of this study showed that the inhibition efficiency of each miRNA embedded in the tri-cistron construction was lower than that for single miRNA. However, the opposite was observed for the native mir-17-92 backbone[29]. These conflicting findings imply that the miRNA tri-cistron can be further optimized to yield more efficient constructs expressing multiple siRNAs against viral replication. The factors that might affect the optimization include the miRNA backbones, the combination, and the linkers. Future studies should investigate the incorporation of other antiviral elements, such as zinc-finger nucleases, single-chain antibodies, and ribozymes, into the linker sequence to produce more antiviral elements from a single miRNA polycistron to inhibit viral infection/escape.

## Conclusions

The tri-cistronic transcript constructed with artificial flanking pri-miRNA sequences simultaneously expresses three anti-HIV siRNAs and efficiently inhibits HIV-1 replication without off-target effects. The strategy described here provides a feasible method to replace the flanking pri-miRNA sequences with other antiviral elements to design more complicated and efficient polycistronic miRNAs.

## Methods

### Plasmid construction

The shRNA expression plasmids were constructed according to the pSUPER instructions provided by the manufacturer (Oligoengine, WA, USA). Target sequences of shRNAs are shown in the Additional file 1: Table S1. The pcDNA3.1 vector was mutated to remove BglII site using QuikChange II Site-Directed Mutagenesis Kits (Agilent, CA, USA). The miRNA expression plasmids were constructed by inserting annealed oligonucleotides (Additional file 1: Table S2), encoding the miRNA target transcript, into the pcDNA3.1 vector at multiple cloning sites (BamHI/EcoRI). The basic elements were obtained by inserting the fragments (EcoRI/XhoI) of the linker (the 3′ end of each linker contains a BglII cloning sites.) into the cloning site (EcoRI/XhoI) of the miRNA-expressing vector. The miRNA cluster was obtained by inserting the fragment (BamHI/XhoI) of one basic structure into the cloning site (BglII/XhoI) of pcDNA3.1 expressing upstream basic structures of the cluster.

An H-2K^k^ expression cassette was cloned from pMACSKK.II (Miltenyi, Bergisch Gladbach, Germany) using primers (5′-TTTACTAGTCATGTTTGACAGCTTATCATCG-3′and 5′-TTTCTCGAGATACAAGGATCCATCTACC CTCCTTTTCCACC-3′). The cleaved fragment (SpeI/XhoI) was inserted into the cloning site (XbaI/XhoI) of pLL3.7 to obtain the pLLK^k^ vector. pCDNA3.1 vectors expressing miRNAs or miRNA clusters were cleaved (BamHI/XhoI) and inserted into the same cleaved pLLK^k^. Fragments containing the RNAi target sequences were generated from pNL4-3 by adding XbaI at 5′ termination site and FseI at 3′ termination site. PCR products were cleaved (XbaI/FseI) and inserted into the cloning site (XbaI/FseI) of the pGL3-control vector (Promega, Madison, WI) to obtain luciferase reporters.

### Cell culture

Human embryonic kidney 293FT adherent cells were purchase from Invitrogen and grown in Dulbecco’s modified Eagle’s medium (Invitrogen, Carlsbad, CA) supplemented with 10% fetal calf serum, penicillin (100 U/mL) and streptomycin (100 μg/mL). MT-4 and TZM-bl cells were obtained from the National Institutes of Health (NIH) AIDS Research and Reference Program and grown in RPMI 1640 medium (Invitrogen) supplemented with 10% fetal calf serum, penicillin (100 U/mL) and streptomycin (100 μg/mL) (complete medium).

### Infectious clone co-transfection experiments

500 ng miRNA expressing vectors (or 500 ng shRNA expressing vectors), 50 ng pRL Renilla Luciferase Control Reporter Vectors (Promega) and 100 ng pNL4-3 were co-transfected into 293FT cells in 24-wells plates at 80% confluency with Lipofectamine 2000 (Invitrogen, Carlsbad, CA). At 48 h post-transfection, CA-p24 levels in the culture supernatant were measured by enzyme-linked immunosorbent assay (ELISA) 48 h post-transfection. The cells were lysed with 120 μL Passive Lysis Buffer (Promega) and luciferase levels were analyzed from 10 μL lysate using the Dual Luciferase reporter assay (50 μL of substrate reagents; Promega) on a Centro LB 960 Microplate Luminometer (Berthold, Bad Wildbad, Germany).

### Reporter co-transfection experiments

300 ng miRNA expressing vectors, 50 ng pRL, and 300 ng luciferase reporters were co-transfected into 293FT cells in 24-well plates with Lipofectamine 2000 (Invitrogen). Cells were lysed to measure luciferase activity 48 h post-transfection. Changes in the expression of firefly luciferase (target) were calculated relative to Renilla luciferase (internal control) and normalized to levels in cells transfected with the pcDNA3.1 control plasmid expressing miR-LacZ.

### Lentiviral vector production and transduction

The pSUPER-Drosha is a shRNA expressing plasmid based on pSUPER vector to decrease the level of Drosha protein (the target sequence is AACGAGUAGGCUUCGUGACUU33). The 293FT cells were seeded in a 10 cm dish (5 × 10^6^/dish). After 6 h, 12 μg pLLK^k^-miRNA vectors were co-transfected with 6 μg pVSVG, 6 μg pMDL, 6 μg pREV, and 6 μg pSUPER-Drosha vectors into 293FT cells using Lipofectamine 2000 reagent (Invitrogen). Culture medium was replaced 12 h post-transfection. On the third day of culture, cell culture supernatant containing lentiviral vectors was harvested and pooled. Cellular debris was removed by filtration through a 0.45 μm filter. Lentiviral stocks were titrated on 293 T cells. MT-4 cells (1 × 10^5^) were transduced with lentivirus expressing miRNA at a multiplicity of infection (MOI) of 40. Ten days post-transduction, cells were sorted with live fluorescence-activated cell sorting (FACS), and green fluorescent protein (GFP)-positive cells were selected.

### miRNA detection by Northern blotting

Total RNA was isolated using Trizol reagent from MT-4 cells or MT-4 cells expressing miRNAs. The miRNAs were detected using a miRNA Northern Blot Assay Kit (Signosis, Sunnyvale, CA), according to the manufacturer’s instructions. Biotinylated probes were used for detection: GTATGTAGGATCTGACTTA (miR-A), GGATTTACCACACCAGACA (miR-B) and GTAGACAGGATGAGGATTA (miR-C).

### MTT cell viability assay

Cells were seeded at a density of 2000/well in 96-well plates and grown three days. MTT (Promega) was added at 15 μL/well and incubated at 37°C for 4 hours. Optical density was measured at 570 nm. All experiments were done in triplicate.

### miRNA detection by quantitative PCR

Total RNA was isolated using Trizol reagent from MT-4 cells or MT-4 cells expressing miRNAs. The miRNAs were detected using a Ncode miRNA qRT-PCR Kit (Invitrogen), according to the manufacturer’s instructions. Sequences of primers are shown in the Additional file 1: Table S3.

### HIV-1 challenge assays

The titer of HIV-1_NL4-3_ was determined by infecting TZM-bl cells and scored for β–galactosidase-positive cells [41]. Briefly, TZM-bl cells were grown in 96-well plates at 1 × 10^4^ cells per well. Cells were infected with 50 μL of 10-fold serially diluted virus. Two days post-infection, cultured cells were fixed and stained. Blue cells with β-galactosidase activity were counted under a light microscope. MT-4 cells or MT-4 cells expressing miRNA were infected by HIV-1 at MOI = 0.1. After 24 h of infection, cells were washed twice with RPMI 1640 medium and cultured in complete medium at 37°C. Viral spread was monitored by measuring CA-p24 production by ELISA.

## Competing interests

The authors declare that they have no competing interests.

## Authors’ contributions

TZ and TC contributed equally to this work, participated in all the laboratory studies, and prepared the manuscript. LW and YC carried out shRNA screening and polycistronic miRNA construction. AY and YY modified the manuscript. JH conceived the idea for the study. JZ and NX critically reviewed and finalized the manuscript. All authors read and approved the final manuscript.

## Supplementary Material

Additional file 1 **Table S1.** shRNA target sequences used in the study. **Table S2.** Oligonucleotide primers used for miRNAs construction. **Table S3.** Primers used for miRNA detection. **Figure S1.** Influence of linkers on the replication activity of HIV-1. For the assays, 300 ng plasmids expressing linkers, 50 ng pRL, and 300 ng pNL4-3 were co-transfected into 293FT. Virus production was measured by CA-p24 ELISA and corrected for transfection efficiency by including Renilla luciferase in the transfection assay. Signal from cells transfected with EGFP expressing plasmid was used as the negative control. **Figure S2.** Structure chart of artificial miRNAs. **Figure S3.** Enhanced inhibition efficiency of HIV-1 by miR-ACB. The miRNA-encoding plasmids based on pLLK^k^ were co-transfected with pNL4-3 in 293FT cells. Virus production was measured by CA-p24 ELISA and corrected for transfection efficiency by including Renilla luciferase in the transfection assay. The signal from the miR-LacZ transfected cells was used as a negative control. Click here for file
