# Supplementary material for: Efficient inhibition of HIV-1 replication by an artificial polycistronic miRNA construct
Source: Virol J. 2012 Jun 18;9:118. doi: 10.1186/1743-422X-9-118 (PMC3416660; doi:10.1186/1743-422X-9-118)

**Additional file 1**

| shRNA | Target sequences | | | shRNA | Target sequences |
| --- | --- | --- | --- | --- | --- |
| Pol1 | AGAAGCAGGAGCCGATAGA | | | Pol41 | CTAATGATGTGAAACAATT |
| Pol2 | AGCCGATAGACAAGGAACT | | | Pol42 | GTGGACAGAGTATTGGCAA |
| Pol3 | AAGACAGTATGATCAGATA | | | Pol43 | TGGACAGAGTATTGGCAAG |
| Pol4 | AGAAATCTGCG GACATAAA | | | Pol44 | GAAGTTATGGTACCAGTTA |
| Pol5 | TCTGCGGACATAAAGCTAT | | | Pol45 | CCCATAATAGGAGCAGAAA |
| Pol6 | CTGCGGACATAAAGCTATA | | | Pol46 | CAGCCAATAGGGAAACTAA |
| Pol7 | AGCTATAGGTACAGTATTA | | | Pol47 | GCCAATAGGGAAACTAAAT |
| Pol8 | CTCAGATTGGCTGCACTTT | | | Pol48 | GCAGGATATGTAACTGACA |
| Pol9 | CAGATTGGCTGCACTTTAA | | | Pol49 | TAACGGACACAACAAATCA |
| Pol10 | CTCAAGATTTCTGGGAAGT | | | Pol50 | CTCACAATATGCATTGGGA |
| Pol11 | CCACATCCTGCAGGGTTAA | | | Pol51 | GGGAATCATTCAAGCACAA |
| Pol12 | AGGAAGTATACTGCATTTA | | | Pol52 | TCAGAGTTAGTCAGTCAAA |
| Pol13 | CAATGAGACACCAGGGATT | | | Pol53 | AGAGTTAGTCAGTCAAATA |
| Pol14 | GAGACACCAGGGATTAGAT | | | Pol54 | CAGTGCTGGAATCAGGAAA |
| Pol15 | GACACCAGGGATTAGATAT | | | Pol55 | GTGCTGGAATCAGGAAAGT |
| Pol16 | TGGAAAGGATCACCAGCAA | | | Pol56 | CCCAAGAAGAACATGAGAA |
| Pol17 | AGGATCACCAGCAATATTC | | | Pol57 | CACAGTAATTGGAGAGCAA |
| Pol18 | CCAGCAATATTCCAGTGTA | | | Pol58 | TAACCTACCACCTGTAGTA |
| Pol19 | GCAATATTCCAGTGTAGCA | | | Pol59 | TACCACCTGTAGTAGCAAA |
| Pol20 | TCCAGTGTAGCATGACAAA | | | Pol60 | TAGTAGCCAGCTGTGATAA |
| Pol21 | CAGACATAGTCATCTATCA | | | Pol61 | CAGGAATATGGCAGCTAGA |
| Pol22 | GTATGTAGGATCTGACTTA | | | Pol62 | GGAATATGGCAGCTAGATT |
| Pol23 | TAGGGCAGCATAGAACAAA | | | Pol63 | TCATGTAGCCAGTGGATAT |
| Pol24 | GAGGAACTGAGACAACATC | | | Pol64 | TGTAGCCAGTGGATATATA |
| Pol25 | GGATTTACCACACCAGACA | | | Pol65 | TGGCAGCAATTTCACCAGT |
| Pol26 | GAAAGAACCTCCATTCCTT | | | Vif1 | GTAGACAGGATGAGGATTA |
| Pol27 | GGGTTATGAACTCCATCCT | | | Vif2 | TAGACAGGATGAGGATTAA |
| Pol28 | TGGACAGTACAGCCTATAG | | | Vif3 | TATTTCAAGGAAAGCTAAG |
| Pol29 | GGACAGTACAGCCTATAGT | | | Vif4 | GACATCACTATGAAAGTAC |
| Pol30 | GCTGGACTGTCAATGACAT | | | Vif5 | CTATGAAAGTACTAATCCA |
| Pol31 | CTGTCAATGACATACAGAA | | | Vif6 | GCATACAGGAGAAAGAGAC |
| Pol32 | GGGATTAAAGTAAGGCAAT | | | Vif7 | GCACACAAGTAGACCCTGA |
| Pol33 | GCACTAACAGAAGTAGTAC | | | Vif8 | CTGACCTAGCAGACCAACT |
| Pol34 | GAGCTAGAACTGGCAGAAA | | | Vif9 | TGACCTAGCAGACCAACTA |
| Pol35 | CCGGTACATGGAGTGTATT | | | Vif10 | ACCTAGCAGACCAACTAAT |
| Pol36 | GGAGTGTATTATGACCCAT | | | Vif11 | CCTAGCAGACCAACTAATT |
| Pol37 | GGCCAATGGACATATCAAA | | | Vif12 | TTTCAGAATCTGCTATAAG |
| Pol38 | CCAATGGACATATCAAATT | | | Vif13 | ATACCATATTAGGACGTAT |
| Pol39 | CCCACACTAATGATGTGAA | | | Vif14 | TACCATATTAGGACGTATA |
| Pol40 | CACTAATGATGTGAAACAA | | | Vif15 | ATTAGGACGTATAGTTAGT |
| shRNA | Target sequences | | | shRNA | Target sequences |
| Vif16 | GGACGTATAGTTAGTCCTA | | | Vif24 | CTTGGCACTAGCAGCATTA |
| Vif17 | ATCAAGCAGGACATAACAA | | | Vif25 | GGCACTAGCAGCATTAATA |
| Vif18 | AGCAGGACATAACAAGGTA | | | Vif26 | GCACTAGCAGCATTAATAA |
| Vif19 | ACATAACAAGGTAGGATCT | | | Vif27 | CACTAGCAGCATTAATAAA |
| Vif20 | GGTAGGATCTCTACAGTAC | | | Vif28 | ACAGATAAAGCCACCTTTG |
| Vif21 | CTCTACAGTACTTGGCACT | | | Vif29 | ATAAAGCCACCTTTGCCTA |
| Vif22 | TCTACAGTACTTGGCACTA | | | Vif30 | CCACCTTTGCCTAGTGTTA |
| Vif23 | CTACAGTACTTGGCACTAG | | |  |  |
|  | |  |  | | |
|  | |  |  | | |

**Additional file 2**

| Name | Nucleotides sequence |
| --- | --- |
| miR-A1f | GATCCGCGC*TTTGTATGTAGGATCTGACTTA*ctgtgaagccacagatgggTAAGTCAGATCCTACATACAAAatgcC |
| miR-A1r | TCGAGgcat*TTTGTATGTAGGATCTGACTTA*cccatctgtggcttcacagTAAGTCAGATCCTACATACAAAGCGCG |
| miR-A2f | GATCCGCGC*TTGTATGTAGGATCTGACTTAG*ctgtgaagccacagatgggCTAAGTCAGATCCTACATACAAatgcC |
| miR-A2r | TCGAGgcat*TTGTATGTAGGATCTGACTTAG*cccatctgtggcttcacagCTAAGTCAGATCCTACATACAAGCGCG |
| miR-A3f | GATCCGCGC*TGTATGTAGGATCTGACTTAGA*ctgtgaagccacagatgggTCTAAGTCAGATCCTACATACAatgcC |
| miR-A3r | TCGAGgcat*TGTATGTAGGATCTGACTTAGA*cccatctgtggcttcacagTCTAAGTCAGATCCTACATACAGCGCG |
| miR-B1f | GATCCgcgc*TGGGGATTTACCACACCAGACA*ctgtgaagccacagatgggTGTCTGGTGTGGTAAATCCCCAatgcC |
| miR-B1r | TCGAGgcat*TGGGGATTTACCACACCAGACA*cccatctgtggcttcacagTGTCTGGTGTGGTAAATCCCCAgcgcG |
| miR-B2f | GATCCgcgc*GGGGATTTACCACACCAGACAA*ctgtgaagccacagatgggTTGTCTGGTGTGGTAAATCCCCatgcC |
| miR-B2r | TCGAGgcat*GGGGATTTACCACACCAGACAA*cccatctgtggcttcacagTTGTCTGGTGTGGTAAATCCCCgcgcG |
| miR-B3f | GATCCgcgc*GGGATTTACCACACCAGACAAA*ctgtgaagccacagatgggTTTGTCTGGTGTGGTAAATCCCatgcC |
| miR-B3r | TCGAGgcat*GGGATTTACCACACCAGACAAA*cccatctgtggcttcacagTTTGTCTGGTGTGGTAAATCCCgcgcG |
| miR-C1f | GATCCTGCTG*AAGTAGACAGGATGAGGATTA*GTTTTGGCCACTGACTGACTAATCCTCATCCTGTCTACTTCAGGAC |
| miR-C1r | TCGAGTCCTG*AAGTAGACAGGATGAGGATTAG*TCAGTCAGTGGCCAAAACTAATCCTCATCCTGTCTACTTCAGCAG |
| miR-C2f | GATCCTGCTG*AGTAGACAGGATGAGGATTAG*GTTTTGGCCACTGACTGACCTAATCCTCATCCTGTCTACTCAGGAC |
| miR-C2r | TCGAGTCCTG*AGTAGACAGGATGAGGATTAGG*TCAGTCAGTGGCCAAAACCTAATCCTCATCCTGTCTACTCAGCAG |
| miR-C3f | GATCCTGCTG*GTAGACAGGATGAGGATTAGA*GTTTTGGCCACTGACTGACTCTAATCCTCATCCTGTCTACCAGGAC |
| miR-C3r | TCGAGTCCTG*GTAGACAGGATGAGGATTAGAG*TCAGTCAGTGGCCAAAACTCTAATCCTCATCCTGTCTACCAGCAG |
| miR-LacZf | GATCCTGCTG*AAATCGCTGATTTGTGTAGTC*GTTTTGGCCACTGACTGACGACTACACATCAGCGATTTCAGGAC |
| miR-LacZr | TCGAGTCCTG*AAATCGCTGATTTGTGTAGTCG*TCAGTCAGTGGCCAAAACGACTACACATCAGCGATTTCAGCAG |

Sequences in italics are RNAi target sequences.

**Additional file 3**

| Primer sequence | Target |
| --- | --- |
| AACATTCAACGCTGTCGGTG | miR-181 |
| TAGCAGCACGTAAATATTGGC | miR-16 |
| ATGACACGCAAATTCGTGAAGC | U6B |

**Additional file 4**


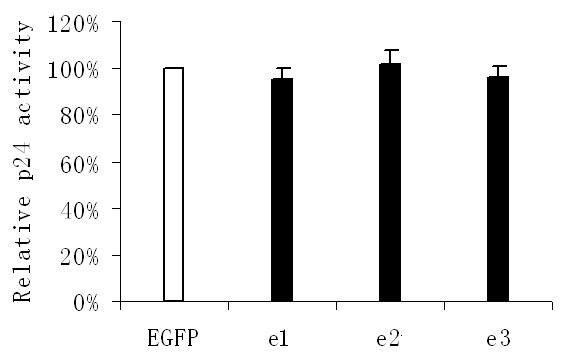


**Additional file 5**

**Additional file 6**


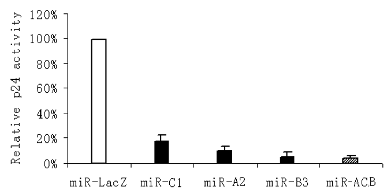

Supplement: Additional file 1 — Table S1. shRNA target sequences used in the study. Table S2. Oligonucleotide primers used for miRNAs construction. Table S3. Primers used for miRNA detection. Figure S1. Influence of linkers on the replication activity of HIV-1. For the assays, 300 ng plasmids expressing linkers, 50 ng pRL, and 300 ng pNL4-3 were co-transfected into 293FT. Virus production was measured by CA-p24 ELISA and corrected for transfection efficiency by including Renilla luciferase in the transfection assay. Signal from cells transfected with EGFP expressing plasmid was used as the negative control. Figure S2. Structure chart of artificial miRNAs. Figure S3. Enhanced inhibition efficiency of HIV-1 by miR-ACB. The miRNA-encoding plasmids based on pLLKk were co-transfected with pNL4-3 in 293FT cells. Virus production was measured by CA-p24 ELISA and corrected for transfection efficiency by including Renilla luciferase in the transfection assay. The signal from the miR-LacZ transfected cells was used as a negative control. [file 1743-422X-9-118-S1.doc]
